# Systemic Effects of Pesticides on Insectivorous Bats: A Proteomics Approach

**DOI:** 10.1093/icb/icaf121

**Published:** 2025-07-01

**Authors:** Natalia Sandoval-Herrera, Linda Lara-Jacobo, Paul A Faure, Denina Simmons, Kenneth Welch

**Affiliations:** Department of Ecology and Evolutionary Biology, University of Toronto, Toronto, ON M5S 3B2, Canada; Department of Biological Sciences, University of Toronto Scarborough, Toronto, ON M1C 1A4, Canada; Department of Wildlife, Fish and Environmental Studies, Swedish University of Agricultural Sciences, Umeå 901 83, Sweden; School of Public Health, San Diego State University, Brawley, CA 92093, USA; Department of Psychology, Neuroscience & Behaviour, McMaster University, Hamilton, ON L8S 4K1, Canada; Faculty of Science, Ontario Tech University, Oshawa, ON L1G 0C5, Canada; Department of Ecology and Evolutionary Biology, University of Toronto, Toronto, ON M5S 3B2, Canada; Department of Biological Sciences, University of Toronto Scarborough, Toronto, ON M1C 1A4, Canada

## Abstract

Bats play a critical role controlling agricultural pests, yet foraging in croplands exposes them to hazardous pesticides. These chemicals pose significant risks for bats by impairing immune function, locomotion, and cognition even at low doses, jeopardizing their survival and ecological role. Here, we employed proteomics—a powerful, yet underused, tool in ecotoxicology—to examine the systemic effects of chlorpyrifos (CPF), a commonly used insecticide, on big brown bats (*Eptesicus fuscus*). We exposed bats through their diet to an environmentally relevant concentration of CPF for three or seven consecutive days and took plasma samples before and after exposure for non-targeted proteomics. We identified over 100 proteins with significant abundance changes before and after exposure to the pesticide. Exposure to CPF altered a wide range of molecular processes, including cell communication, cell metabolism, and DNA maintenance. Remarkably, we found changes in key proteins involved in immune response, T cell activation, and inflammation. These effects could reduce a bat's immune response, increasing their susceptibility to viral infections, and intensifying the risk of shedding and transmitting pathogens to other species. Our results provide new insights into the toxicity of pesticides and highlight the utility of proteomics for assessing toxicant effects in understudied and vulnerable species such as bats. Considering a One Health approach and the role of bats as reservoirs for numerous zoonotic pathogens, our work has broad implications for bat and human health.

## Introduction

Pesticide use associated with agricultural intensification poses significant threats to wildlife, particularly non-target species such as bats. Insectivorous bats, for example, provide essential pest control services by foraging in agricultural landscapes, yet this behavior also exposes them to hazardous agrochemicals present in the air, water, and prey (Stahlschmidt et al. 2017; Hernández‐Jerez et al. 2019). While both acute and lethal effects of pesticide exposure on bats are documented (Humphrey and Cope 1977; Clark 1981; Mitchell‐Jones et al. 1989), sublethal physiological impacts remain poorly understood (Torquetti et al. 2021), despite being more likely at environmentally relevant concentrations (Bayat et al. 2014; Williams-Guillén et al. 2015). Recent research suggests that pesticides can disrupt processes at multiple levels of biological organization (cells to behavior), leading to immunotoxicity, endocrine disruption, neurotoxicity, and chronic stress (reviewed in Oliveira et al. 2020; Torquetti et al. 2021). These effects may compromise individual bat health and survival and, over time, threaten bat populations (O'Shea and Clark 2002; Bayat et al. 2014; Torquetti et al. 2021).

Among sublethal effects, disruption in immune system function is particularly concerning, given its potential to increase disease susceptibility and alter pathogen dynamics. Bats serve as natural reservoirs for numerous zoonotic viruses transmitted to humans, including Ebola virus, Nipah virus, SARS-CoV-1, and SARS-CoV-2 (Calisher et al. 2006; Schountz 2014; Han et al. 2015; Wang and Anderson 2019). Unlike many mammals, bats possess unique immune adaptations that allow them to harbor viruses without developing evident disease symptoms (Banerjee et al. 2020; Wang et al. 2021; Demian et al. 2024). However, environmental stressors such as pesticide exposure may compromise these defenses, leading to greater susceptibility to infection or increased disease-induced mortality. Alternatively, sublethal toxicant effects could enhance viral shedding and transmission even while symptoms remain mild or absent (tolerance), as observed in other vertebrates (Caren 1981; Hayes et al. 2010; Kelly et al. 2010; Desforges et al. 2016; Sanderfoot and Letters 2017). For example, physiological chronic stress has been proposed as a main driver of viral excretion cycles in bats by reactivating latent viruses (Plowright et al. 2016). Understanding how anthropogenic stressors affect bat immune competence is therefore critical, not only for bat conservation but also in the context of One Health (Mackenzie and Jeggo 2019) to elucidate unknown implications for disease ecology and public health.

Pesticide exposure can affect multiple physiological mechanisms, leading to a wide range of impacts at the individual level, which makes studying its consequences a complex and challenging task. Although concerns about the implications for bat immunity have been raised and ecological consequences discussed (Mason 2013; Hooper and Amelon 2014; Plowright et al. 2015; Sánchez et al. 2020; Becker et al. 2024), the direct mechanisms through which pesticides influence bat immunity are largely unknown. Given the diversity of their modes of action and routes of exposure, pesticides can alter immune function through multiple pathways. Some of the best-characterized mechanisms in mammals include changes in cytokine production, elevated glucocorticoid levels, reduced white blood cell counts, and the induction of oxidative stress (Grasman 2002; Galloway and Handy 2003; Descotes 2004). These alterations can manifest at the individual level as immune suppression, hypersensitivity, or autoimmunity (Bou Zerdan et al. 2021). Research on contaminants and immunity in bats is scarce and has largely focused on the effects of heavy metals on single biomarkers such as leukocyte counts, microbicidal assays, oxidative stress markers, and micronucleus assays (Lilley et al. 2013; Pilosof et al. 2014; Becker et al. 2017; Costantini et al. 2019; Sandoval-Herrera et al. 2021). While these targeted techniques provide valuable insights into the known immunotoxic effects across various taxa, they captured only a fraction of the physiological processes impacted by these stressors. To get a better understanding of the effects, these traditional biomarker-based approaches can be complemented by high-throughput approaches such as untargeted omics, which can reveal previously unrecognized pathways and mechanisms through which contaminants impact wildlife physiology.

Proteomics offers a powerful and comprehensive method to examine how toxicants simultaneously affect multiple molecular processes from a single sample and in a particular context (Snape et al. 2004; Porreca et al. 2014). These methods enable the identification of broad-scale protein abundance changes, providing insights into metabolism, cellular signaling, immune function, and gene regulation (Gouveia et al. 2019; Liang et al. 2020). This system-level approach is particularly valuable for studying the effects of environmental contaminants in wildlife, where the lack of baseline physiological information hinders the utility of traditional toxicological assessments (Garcia-Reyero and Perkins 2011; Martyniuk and Simmons 2016). Furthermore, proteomics facilitates biomarker discovery, offering early-warning indicators of toxicity and exposure, which can be used to develop new endpoints for chemical risk assessment in free-living organisms. Blood-based proteomics is particularly well suited for bat ecotoxicology, as it allows for the analysis of systemic responses to toxicants using minimally invasive sampling (e.g., <10 μL of blood) and with fewer animals (Langan 2022).

Considering the knowledge gap on the effects of pesticides in bats and the advantages provided by current throughput methods, here we used proteomics to evaluate the cellular and physiological effects of pesticide exposure in the big brown bat (*Eptesicus fuscus*) as a model insectivorous bat species. We examined the systemic effects of pesticide exposure by analyzing changes in the plasma proteome following exposure to an environmentally relevant dose of chlorpyrifos (CPF), a commonly used insecticide. As an organophosphate pesticide, CPF is primarily known for its neurotoxic effects; it inhibits acetylcholinesterase (AChE), an enzyme critical for nerve signal transmission (Gupta 2006; Eaton et al. 2008). We expected to detect significant changes in the plasma proteome of bats following CPF exposure reflecting both coping mechanisms and pesticide toxicity. Specifically, we predicted to see alterations in proteins associated with nervous system function and oxidative stress, mechanisms that have been widely reported for CPF toxicity (Gupta 2006), and that can indirectly affect immune function. Additionally, we expect to see changes in proteins directly related to immune functions such as inflammation and leukocyte function, effects that have been previously reported in mammals. Furthermore, we anticipated differences in proteomic responses between bats exposed for 3 and 7 days, potentially reflecting time-dependent physiological adaptations and/or cumulative toxic effects. Here, we present the systemic effects of CPF on the plasma proteome, with a focused discussion on changes in proteins linked to immune-related pathways. We chose to emphasize immune function due to the limited understanding of these mechanisms and their relevance in assessing how chemical stressors may impact bat health, an important consideration for both wildlife conservation and public health. Our study highlights the value of proteomics in wildlife toxicology and provides novel insights into the mechanisms by which environmental contaminants affect bats.

## Materials and methods

### Experimental animals and housing

We used big brown bats (*Eptesicus fuscus*) from a captive research colony established at McMaster University in Hamilton, Ontario, Canada. Bats were either wild caught in Southern Ontario or direct descendants of wild caught individuals. Bats were housed indoors (2.5 × 1.5 × 2.3 m; l × w × h), in a mixed-sex colony where the temperature and lighting varied with ambient conditions, with access to a larger, outdoor flying area (2.5 × 3.8 × 2.7 m) with tree branches and hanging vines as habitat enrichment (Skrinyer et al. 2017). Bats had *ad libitum* access to water and food (yellow mealworms *Tenebrio molitor*; Reptile Feeders, Norfolk, ON), and were provided with natural (hollowed tree with bark) and artificial (folded towels) roosts. All bats selected for experiments were adult females that had lived in the colony for at least 6 months (*n* = 18, 9 in 2019 and 9 in 2020). During experiments, bats were kept overnight in stainless steel wire mesh cages (28 × 22 × 18 cm, ¼″ mesh) in an indoor holding room. Bats were housed individually so they could be video-monitored before exposure and during exposure. Behavioral and neurological changes were evaluated *in situ* at different time points.

All procedures were approved by the Animal Research Ethics Board of McMaster University (AUP #16–06-25 and #20–05-20) and conformed to the Guidelines for the Care and Use of Experimental Animals in Research published by the Canadian Council on Animal Care.

### Pesticide exposure

Bats were orally exposed (details below) to the same concentration of the insecticide CPF over two different durations. One group (*n* = 6) in 2019 was exposed to CPF daily for three consecutive days (CPF-3d), while another group (*n* = 6) in 2020 underwent CPF exposure for seven consecutive days (CPF-7d). A control group (*n*_2019_ = 3, *n*_2020_ = 3) received a sham treatment. This exposure regimen was designed to mimic environmentally realistic conditions of sub-acute exposure, reflecting peak pesticide application levels in both small and large plantations.

In both experimental groups, bats were dosed by feeding them mealworms (*Tenebrio molitor*) injected with a CPF solution. Control group bats were fed mealworms injected only with the oil vehicle (i.e., sham treatment). Experimental bats received a pesticide dose of 10 μg/g of body weight (BW) per day, administered at the same time each evening (1800 h). This dose was based on estimated daily intake calculations derived from CPF concentrations detected in arthropods collected from agricultural fields and the approximate ingestion rate normalized by BW (US EPA 1992; Solomon et al. 2010). It falls within the lower range of concentrations that an insectivorous bat might realistically ingest while foraging in croplands. It also corresponds to the benchmark CPF dose (BMD10), which represents the central estimate for a 10% increase in response, reported to alter 10% of plasma cholinesterase (ChE) activity—a key neurotoxicity biomarker—in big brown bats (Eidels et al. 2016).

### Blood collection

We collected two blood samples from each bat: one before experimental (or sham) exposure (BE), and one 3 or 7 days after exposure (AE). Each sample was a maximum of 50 μL of blood (< 1% of BW), obtained via venipuncture of the cephalic artery located in the propatagium. To aid recovery of electrolytes and fluid volume, bats received Ringer's solution post-blood collection. A small drop of blood was used to prepare blood smears, while the remainder was placed in heparinized 0.5-mL tubes and centrifuged at 2000 x *g*for 5 min. The separated plasma was then transferred to a new tube and stored at -80°C until further analysis.

### Plasma proteomics

We first quantified protein concentration in the plasma samples using a Bradford assay (Bradford 1976). We transferred 5 μL of plasma to a low-retention micro centrifuge tube with 35 μL of 100 mM ammonium bicarbonate (AB) Buffer and mixed with gentle vortex. We then reduced the proteins in the plasma with the addition of 2.65 μL of 100 mM tris(2-carboxyethyl) phosphine, in 100 mM AB buffer, mixed using gentle vortex, and allowed to incubate at room temperature for 45 min. Proteins were then alkylated with the addition of 2.8 μL of 200 mM iodoacetamide in 100 mM AB buffer, vortexed gently, and incubated in the dark at room temperature for 45 min. At the end of the second incubation, 50 μL of chemical digestion solution (20% formic acid v/v) was added to each sample and vortexed for 5 s. Lid locks were placed on each tube and they were incubated at 115°C for 30 min (VWR model 96 place heating block). Samples were dried in a centrifugal evaporator (SpeedVac, Thermo Fisher) for 40 min, stored at 4°C overnight, and then resuspended in 20 μL of a solution of 95% H_2_O, 5% acetonitrile, and 0.1% formic acid. Samples were vortexed until the dried pellets were completely dissolved, and then centrifuged for 10 min at 14,000 *g*. A 20 μL sample of the supernatant and 1 μL of internal peptide standard (H2016, Sigma–Aldrich, Oakville, ON) were added to a 2 mL threaded HPLC (High-Performance Liquid Chromatography) screw vial (Chromatographic Specialties, 12 × 32 mm) containing 250 μL PP (Polypropylene) bottom spring inserts (Canadian Life Sciences, 6 × 29 mm). Samples were stored at 4°C until instrumental analysis.

A 2 μL aliquot of the peptide solution from each sample was injected and then separated by reverse phase liquid chromatography using a Zorbax, 300SB-C18, 1.0 × 50 mm 3.5 μm column (Agilent Technologies Canada Inc., Mississauga, ON) and Agilent 1260 Infinity Binary liquid chromatographer. The Agilent 6545 Accurate-Mass Quadrupole-Time-of Flight (Q-TOF) was used as the detector in tandem to the Agilent 1200 series liquid chromatography system (see Supplementary Appendix I for detailed instrumental methods).

Each analytical run included a solvent blank and a bovine serum albumin (BSA) digest standard (Agilent Technologies Canada Inc., Mississauga, ON) injection every 10 samples to monitor baseline, carry-over, drift, and sensitivity during the runtime. As technical replicates we injected each sample twice and selected the highest value between the two.

Spectral files for each sample were analyzed using Spectrum Mill software (version B.04.01.141). Given the limited entries available for *E. fuscus*, peptides were searched against the Uniprot Reference Proteome of the bat family Vespertilionidae (ID 9431150920 proteins; downloaded April 2021). Proteins were manually validated and accepted when at least one peptide had a score (quality of the raw match between the observed spectrum and the theoretical spectrum) greater than 5 and a %SPI (Spectral Index, % of the spectral intensity accounted for by theoretical fragments) greater than 60%; both are manufacturer-recommended settings for validating results with an Agilent Q-TOF mass spectrometer. After peptides were sequenced and identified by Spectrum Mill at the tandem mass spectrometry (MS/MS) level, quantification at the precursor intensity (MS1) level was performed using the data-dependent acquisition (DDA) workflow in Skyline 20.2 (MacCoss Lab Software) with a score of 0.9, retention time window of 5 min, and 5 missed cleavages with transition settings for TOF (Pino et al. 2020).

### Statistical analysis

Proteomics data were sorted and manually consolidated on a spreadsheet (Microsoft Excel) and statistical analyses were performed with MetaboAnalyst 5.0 (Chong et al. 2019). Missing values were replaced with one-fifth of the limit of detection (LOD) and data were normalized using median, Pareto scaling, and log transformed. We used the normalized-transformed data and compared the treatments with a repeated measures ANOVA [Fisher's least significant difference post-hoc test with Benjamini–Hochberg (B–H) false discovery rate (FDR = 0.25) and partial least-squares discriminant analyses]. The model included condition (BE–AE) as a within-subject factor. Sphericity was tested within ANOVA test function in the *rstatix* (Kassambara 2023) package in R (v.4.3.0), which uses Mauchly's test and automatically applies the Greenhouse–Geisser sphericity correction only to within-subject factors violating the sphericity assumption. We analyzed 36 samples of plasma, with 2 samples per individual: 1 BE and 1 AE. We separated the statistical analysis by exposure duration (year). Last, we used the Revigo server to consolidate and visualize the gene ontology (GO) enriched pathways (Supek et al. 2011). For biomarker diagnostics, we assessed the specificity (false positive rate) and sensitivity (true positive rate) of potential biomarkers using the area under the curve (AUC) of the receiver operator characteristic (ROC) as a measure of how well a parameter distinguished between the two diagnostic groups: BE and AE to CPF, we used a threshold of 0.8 to classify a protein as potential biomarker and a linear support vector machine as classification method. We used the multivariate exploratory ROC analysis in MetaboAnalyst, which uses repeated, balanced sub-sampling Monte Carlo cross-validation (MCCV) to test the performance of classifier models created with different numbers of proteins. As results, we showed that the proteins highly ranked in the best performing classification model and most frequently selected across models.

## Results

### CPF exposure for 3 days

**Fig. 1:**
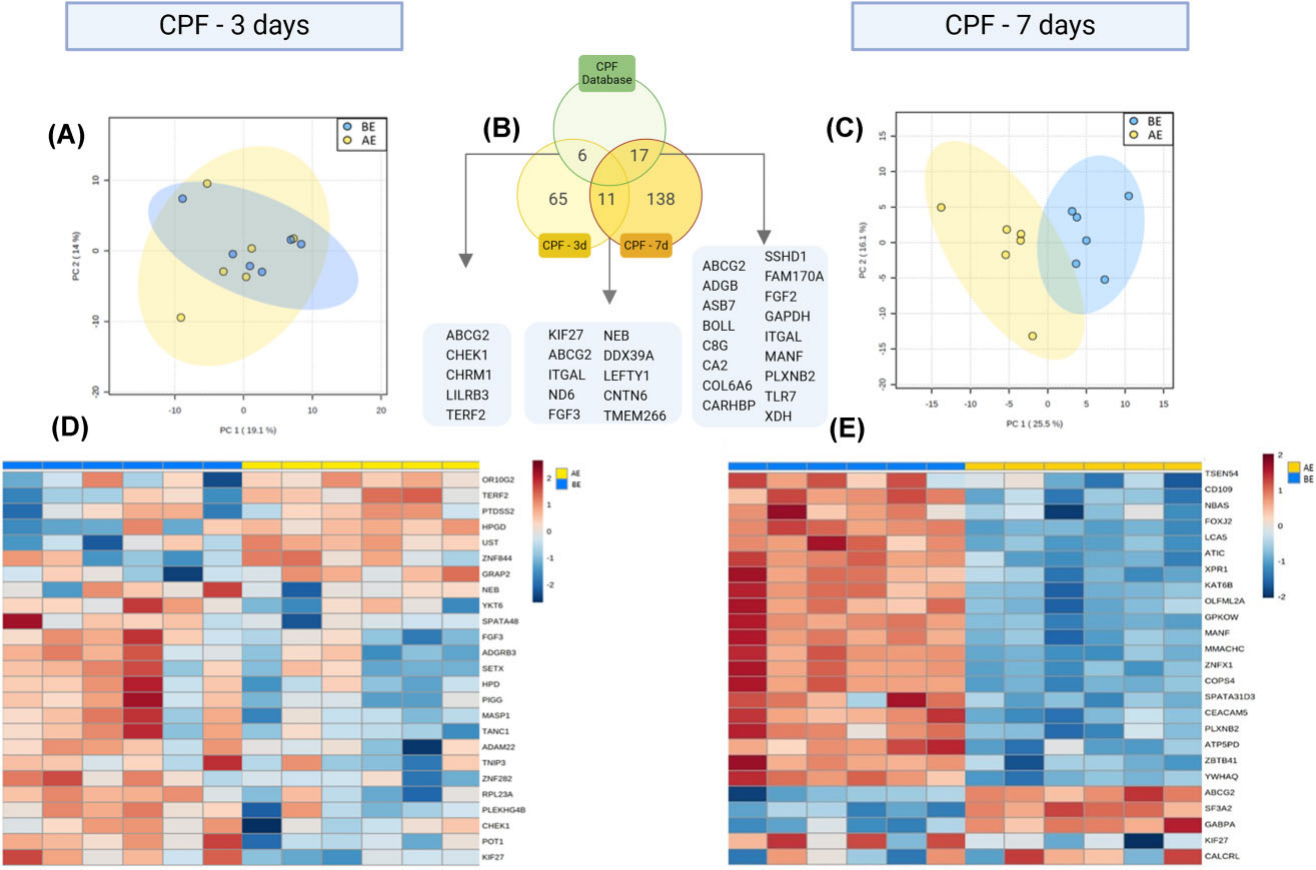
Differential expression of proteins in plasma samples of bats exposed to the insecticide CPF, for three (CPF-3d, left panel) and seven (CPF-7d, right panel) consecutive days. (A, C) Scores plot of principal component analysis showing grouping of the condition BE and AE for both treatments. The explained variances are shown in brackets. (B) Venn diagram showing the breakdown and number of deregulated proteins shared between treatments and with reports of CPF toxicity in the comparative toxicogenomic database (green). (D, E) Heatmaps showing the differential expression of the top 25 proteins differentially expressed (ANOVA *P* > 0.1) in both treatments.

We detected a total of 760 proteins in plasma samples of bats exposed for 3 days (Fig. 1). When comparing the proteomic profile among conditions (BE vs AE), we found 65 differentially abundant proteins [DAPs; fold change (FC) > 1; *P* < 0.1], with 28 upregulated and 37 downregulated (Fig. 1A, D). None of these proteins remained significantly different after B–H correction. The most upregulated proteins included TERF2 (telomeric repeat-binding factor 2; FC = 1.8; *P* = 0.003) and UST (uronyl 2-sulfotransferase; FC = 2.4; *P* = 0.03), suggesting CPF-induced effects on telomere maintenance and cellular apoptosis. Conversely, KIF27 (kinesin family member 27; FC = 0.4; *P* = 0.01) and POT1 (protection of telomeres 1; FC = 0.5; *P* = 0.004) were the most downregulated, indicating potential disruptions in cytoskeletal dynamics and genomic stability. We observed downregulation in proteins associated with energetic metabolism like mitochondrial function, ATP5PD (ATP synthesis) and ND6 (cellular respiration). Similarly, we detected changes in proteins associated with neural function, including CNTN6, LEFTY1, and PCDHGA2—key regulators of neural connectivity and development. A summary of the five most downregulated and five most upregulated (based on the fold change) proteins and their functions is provided in Table 1.

**Table 1:** Main differentially abundant proteins in the plasma of bats exposed to the insecticide chlorpyrifos for three consecutive days.

	Gene	Protein name	FC	*P*-value	Protein family/type	Function
Downregulated	KIF27	Kinesin-like protein KIF27	0.379	0.012	Microtubule binding motor protein	Motile ciliogenesis
	POT1	Protection of telomeres protein 1	0.529	0.004	DNA metabolism protein	Telomer maintenance. negative regulator of telomerase activity
	HPD	4-hydroxyphenylpyruvate dioxygenase	0.695	0.013	Oxygenase	Degradation of tyrosine
	ZNF282	Zinc finger protein 282	0.698	0.007	Zinc finger transcription factor	DNA-binding transcription factor activity; negative regulation
	ADAM22	Disintegrin and metalloproteinase domain-containing protein 22	0.715	0.008	Metalloprotease	Involved in regulation of cell adhesion and spreading and in inhibition of cell proliferation
	MASP1	Mannan-binding lectin serine protease 1	0.750	0.010	Serine protease	Component of the lectin pathway of complement activation
Upregulated	SNX33	Sorting nexin-33	8.345	0.054	Scaffold/adaptor protein	Reorganization of the cytoskeleton, endocytosis, and cellular vesicle trafficking
	UST	Uronyl 2-sulfotransferase	2.458	0.034	Transferase	Transfer of sulfate to the position 2 of uronyl residues, for example, in dermatan sulfate
	HPGD	15-hydroxyprostaglandin dehydrogenase [NAD(+)]	2.016	0.026	Dehydrogenase	Prostaglandin inactivation; regulate inflammation. Apoptosis
	TERF2	Telomeric repeat-binding factor 2	1.837	0.002	Nucleoprotein	Telomer protection; negative regulator of telomere length
	GRAP2	GRB2-related adapter protein 2	1.511	0.044	Adaptor-like protein	Leukocyte-specific protein-tyrosine kinase signaling regulate NF-AT activation
	PTDSS2	Phosphatidylserine synthase 2	1.222	0.034	Transferase	Conversion of phosphatidylethanolamine to phosphatidylserine, a structural membrane phospholipid

FC: fold change.

**Fig. 2:**
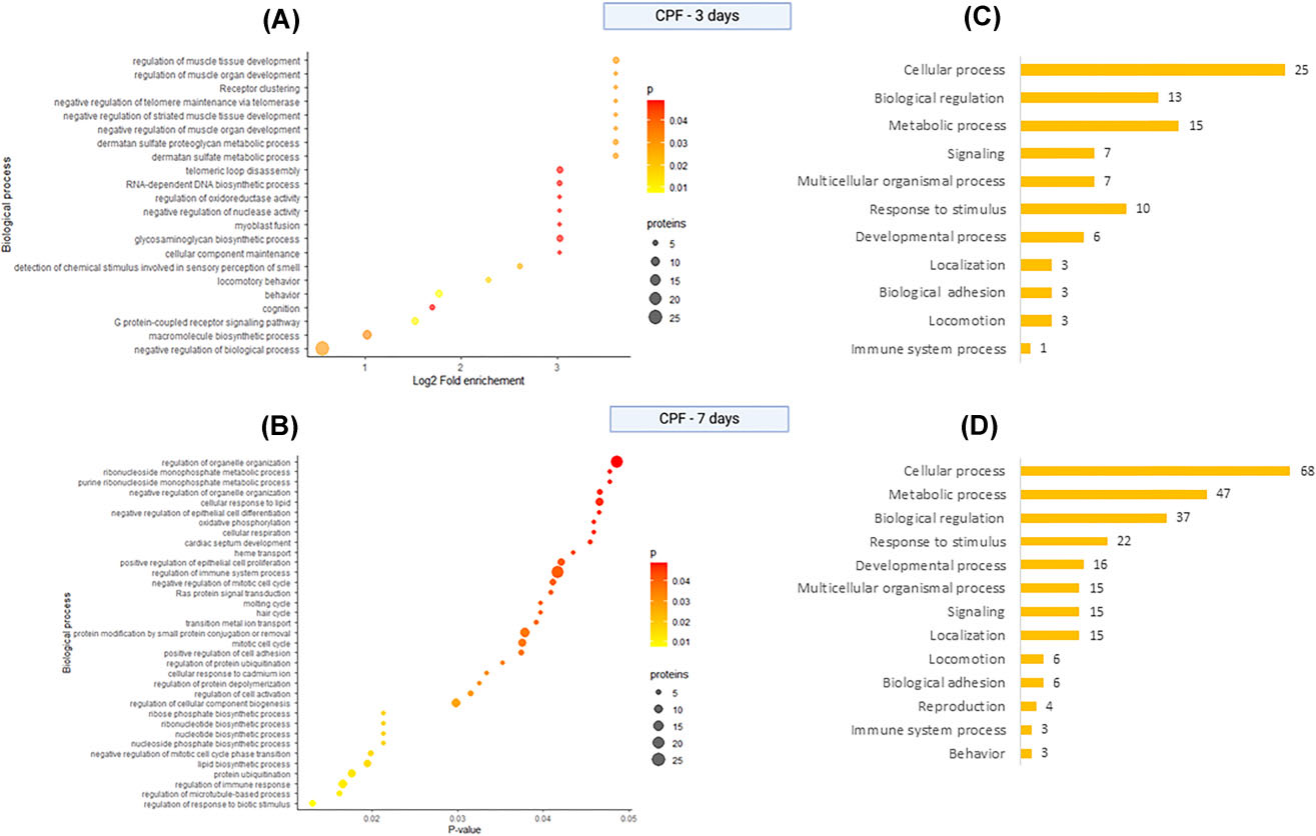
Enrichment analysis for plasma proteome of bats exposed to CPF for three (CPF-3d) and seven (CPF-7d) consecutive days. (A, B) Biological processes enriched in bats exposed to CPF. Size of the bubble shows the number of proteins representing each process, and the color the corresponding *P*-value. (C, D) Bar plot showing a simplified version of the main biological processes represented by the DAPs found in plasma samples of bats exposed to CPF for seven consecutive days. The numbers correspond to the number of DAPs involved in each process.

Functional pathway enrichment analysis revealed that most DAPs were associated with cellular metabolism, DNA maintenance, cellular stress responses, and immune function (Fig. 2A, B). Several biological processes were significantly enriched (*P* < 0.05), highlighting early CPF-induced physiological disruptions. We found that six DAPs had been previously linked to CPF toxicity in the Comparative Toxicogenomics Database (CTD) (Davis et al. 2025), reinforcing their relevance in pesticide-related toxicity. These included proteins involved in DNA damage response (CHEK1, TERF2), neurotransmission (CHRM1), immune regulation (LILRB3), and oxidative stress (HPD).

### CPF exposure for 7 days

We detected a similar number of plasma proteins in bats exposed to CPF for 7 days (761). We found 218 DAP between pre- and post-exposure conditions (FC > 1; *P* < 0.1), of which 138 remained statistically significant after B–H correction. Among these, 57 proteins were upregulated, while 81 were downregulated (Fig. 1 C, E). The most upregulated proteins included CYTB (cytochrome b; FC = 8), a key component of mitochondrial function and oxidative phosphorylation, and SMARCD2 (SWI/SNF-related matrix-associated actin-dependent regulator of chromatin subfamily D member 2; FC = 2), involved in chromatin remodeling and immune cell differentiation. Conversely, the most downregulated proteins were RPS13 (ribosomal protein S13; FC = 0.09), essential for ribosomal assembly and protein synthesis, and SHISA9 (Shisa family member 9; FC = 0.13), which plays a role in synaptic function. On the other hand, the upregulation of proteins involved in xenobiotic detoxification (UGT2A3) and glycolysis (GAPDH) shows evidence of cellular coping mechanisms. A summary of the top five most downregulated and five most upregulated proteins and their functions is provided in Table 2. The functional enrichment analysis identified significant disruptions in biological processes related to metabolism, immune regulation, and cellular stress responses (Fig. 2C, D). Remarkably, 21 (15%) of the DAPs were linked to immune response processes according to the GO panther database (Table 3). Comparing the 3- and 7-day exposure groups, 11 proteins were differentially expressed in both conditions, indicating sustained molecular alterations over time (Fig. 1B).

**Table 2:** Main differentially abundant proteins in plasma of bats exposed to a low dose of the insecticide chlorpyrifos for seven consecutive days.

	Gene	Protein name	FC	*P*-value	Protein family/type	Function
Downregulated	FTL	Ferritin light chain	0.15177	0.053	Membrane trafficking	Involved in iron storage and homeostasis, protecting cells from oxidative damage
	LIMS1	LIM zinc finger domain-containing protein 1	0.13559	0.030	Protease inhibitor	Plays a role in cell adhesion, cytoskeleton organization, and mechanotransduction
	DDX52	DEAD-Box Helicase 52	0.13132	0.011	Protease inhibitor	Functions in ribosome biogenesis, RNA processing, and translation regulation
	SHISA9	Shisa family member 9	0.12721	0.038	Ribosomal protein	Modulates AMPA receptor activity and synaptic transmission, influencing neuronal signaling
	RPS13	Ribosomal protein S13	0.0917	0.005	Cell junction protein	A structural component of the ribosome, essential for protein synthesis and cellular growth
Upregulated	cytb	Cytochrome b	8.6482	0.004		Involved in the electron transport chain and mitochondrial respiration, essential for ATP production
	SMARCD2	SWI/SNF-related matrix-associated actin-dependent regulator of chromatin subfamily D member 2	4.7275	0.005	Storage protein	Regulates chromatin structure and transcription, playing a role in hematopoiesis and immune cell differentiation
	UGT2A3	UDP-glucuronosyltransferase 2A3	4.4582	0.013		Catalyzes glucuronidation, a detoxification process that makes lipophilic compounds more water-soluble for excretion
	ENTHD1	ENTH domain containing 1	3.9714	0.072	Chromatin-binding	Involved in membrane trafficking and endocytosis, potentially influencing cellular signaling
	HRG	Histidine-rich glycoprotein	3.7997	0.084	RNA helicase	Regulates immune response, angiogenesis, and coagulation; interacts with immune cells and modulates inflammation

FC: fold change.

**Table 3:** Differentially abundant proteins involved in immune function pathways in plasma of bats exposed to insecticide chlorpyrifos for 3 (CPF-3d) and 7 days (CPF-7d).

Protein	Protein name	FC CPF-3d		FC CPF-7d		Immune-related function
BST2	Bone marrow stromal antigen 2 (tetherin)	0.40	**↑**	−2.10	**↓**	Inhibits release of enveloped viruses from infected cells
MSH6	DNA mismatch repair protein Msh6	0.41	**↑**	−2.03	**↓**	Indirectly impacts immune response through genomic stability in immune cells
ZNFX1	NFX1-type zinc finger-containing protein 1	−0.16	**↓**	−1.96	**↓**	Involved in sensing viral RNA and type I interferon responses
A1BG	Alpha-1B-glycoprotein	0.31	**↑**	−1.90	**↓**	Immunoglobulin with function unclear expected to participate in recognition and cell adhesion in the immune system; cancer biomarker
CD99L2	CD99 antigen-like protein 2	−0.58	**↓**	−1.67	**↓**	Involved in leukocyte extravasation and immune cell transmigration through endothelial barriers
SDC1	Syndecan-1	−0.37	**↓**	−1.53	**↓**	Mediates cell–cell adhesion, inflammation, and wound healing in immune responses
ITGAL	Integrin alpha-L	0.46	**↑**	−1.15	**↓**	Involved in leukocyte adhesion and migration, essential for immune surveillance
CD109	Activated T-Cell marker CD109	−0.45	**↓**	−1.03	**↓**	Negatively regulates TGF-β signaling, affecting immune cell differentiation
DDX39A	ATP-dependent RNA helicase DDX39A	−1.57	**↓**	−0.91	**↓**	Associated with RNA processing and innate immune signaling, including type I IFN response
EPCAM	Epithelial cell adhesion molecule	−0.19	**↓**	−0.68	**↓**	Involved in epithelial integrity, immune surveillance, and inflammation
NFKBIE	NF-Kappa-B inhibitorepsilon	−0.12	**↓**	−0.52	**↓**	Regulates NF-κB pathway, crucial for inflammation and immune response
TNXB	Tenascin XB	0.00	**↔**	0.58	**↑**	Extracellular matrix protein with minor immune implications, possibly in autoimmunity or tissue repair
IKZF1	DNA-binding protein Ikaros	−0.10	**↓**	0.68	**↑**	Regulates lymphocyte development and immune cell differentiation
C8G	Complement component C8 gamma chain	−0.04	**↓**	0.74	**↑**	Component of membrane attack complex (MAC) for pathogen lysis
FGF2	Fibroblast growth factor 2	0.13	**↑**	0.84	**↑**	Modulates inflammation and tissue regeneration, influencing immune environment
TLR7	Toll-like receptor 7	−0.42	**↓**	0.96	**↑**	Detects viral RNA, activates innate immune response
BPI	Bactericidal permeability-increasing protein	−0.55	**↓**	1.38	**↑**	Binds to LPS of Gram-negative bacteria, aiding in their clearance
CD34	Hematopoietic progenitor cell antigen CD34	−0.45	**↓**	1.51	**↑**	Involved in early stages of immune cell development
GAPDH	Glyceraldehyde-3-phosphate dehydrogenase	0.48	**↑**	1.58	**↑**	Multifunctional enzyme involved in immune regulation and inflammatory signaling under stress
GZMB	Granzyme B	0.21	**↑**	1.63	**↑**	Induces apoptosis in virus-infected and cancerous cells, secreted by cytotoxic T and NK cells
GIMAP8	Immune-associated nucleotide-binding protein 9	1.54	**↑**	1.68	**↑**	GTPase involved in T cell survival and lymphocyte development

FC: log2 fold change.

**Fig. 3:**
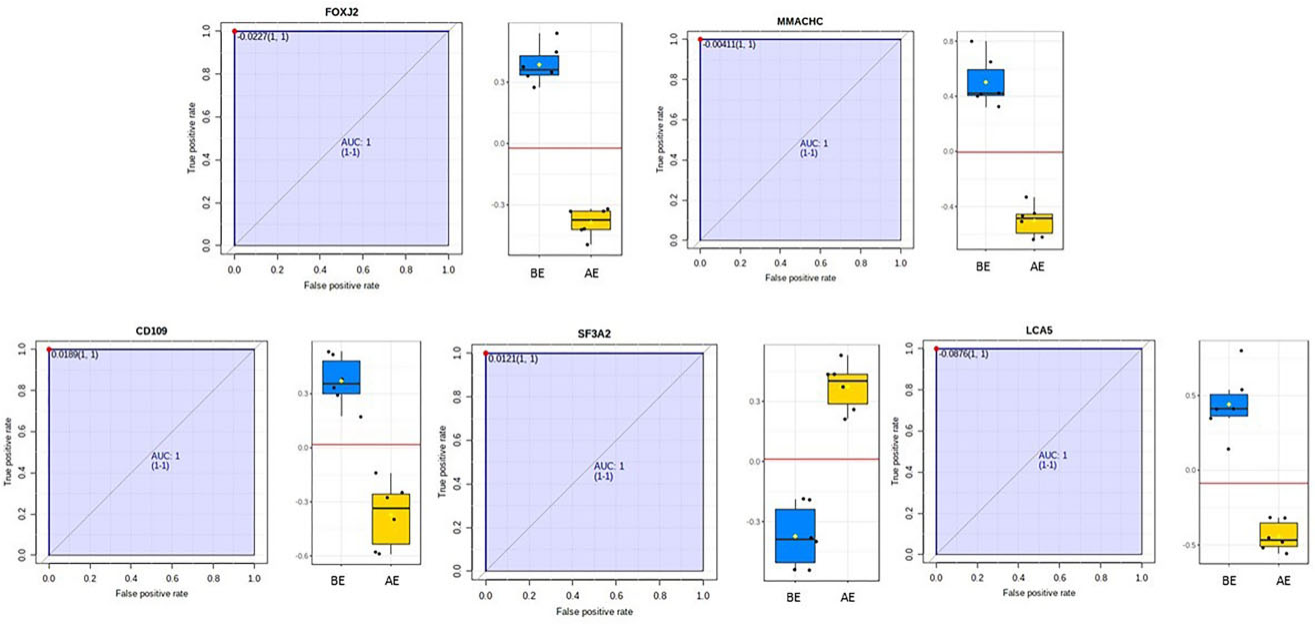
ROC curves showing the sensitivity and specificity of five proteins as biomarkers. AUC values greater than 0.9 indicate a good predictability of the protein to discriminate between conditions (BE–AE). The boxplots on the right-hand side of the curve show the normalized protein concentration. The horizontal line and number in the top right-hand corner indicate the discrimination threshold where the sensitivity was the highest.

Several proteins showed strong potential as biomarkers of CPF exposure based on their ROC curve performance (AUC = 1.0; *P* < 0.01) (Fig. 3). The average accuracy for discrimination was 0.963 based on 100 cross-validations. The best-performing classification model included 15 proteins. The top five candidates included FOXJ2 and CD109, both regulators of cell division and differentiation; MMACHC, a key player in cellular metabolism; SF3A2, a splicing factor involved in RNA synthesis and modification, which was notably downregulated and has been identified as a negative regulator of immune function (De Arras and Alper 2013); and LCA5, which regulates cell migration.

## Discussion

Bats and their remarkable immune systems have garnered significant attention over the past decade, particularly due to their role as reservoirs for numerous pathogens (reviews in Solomon et al. 2010; Banerjee et al. 2020; Wang et al. 2021). However, our understanding of how environmental stressors disrupt their immune function—and the broader implications for both bat and human health—remains limited. In this study, we employed proteomics, a cutting-edge technique, to investigate the systemic effects of pesticides on bat physiology, with a particular focus on immune function. Our findings revealed significant alterations in protein abundance, highlighting the widespread molecular disruptions caused by CPF, even at environmentally relevant concentrations. These disruptions affect fundamental biological processes, including immune response, cell communication, and DNA maintenance, all of which are crucial for the health and resilience of bats. The observed proteome changes related to immune function raise serious concerns about a potential pesticide-induced increased susceptibility to pathogens, which could threaten bat populations and their ecosystem function. Our study provides novel insights into the toxicological effects of pesticides on non-target species and underscores the importance of incorporating high-throughput methodologies such as proteomics into ecotoxicological assessments, especially for understudied and vulnerable taxa.

CPF is one of the most widely used insecticides globally, applied to crops such as corn and soybeans, as well as in managed landscapes such as golf courses and greenhouses (Smegal (UEPA) 2000; Foong et al. 2020). Despite its ban in the USA and Europe, CPF remains extensively used in agriculture-heavy and biodiverse regions of the Global South (Wołejko et al. 2022), posing a significant threat to wildlife. In bats, CPF has been shown to induce genotoxic effects, trigger oxidative stress, and alter immune cell counts (Eidels et al. 2016; Sandoval-Herrera et al. 2023). Its neurotoxicity has been associated with impaired reflex responses and disrupted navigation (Eidels et al. 2016; Sandoval-Herrera et al. 2022). We identified several proteins previously associated with CPF exposure, reinforcing its broad physiological impact and revealing significant cellular responses even at low concentrations and short exposure durations. Our findings build on these previous reports by providing a mechanistic perspective on CPF toxicity at the proteomic level. Some of these changes align with expected detoxification pathways, such as upregulation of xenobiotic transporters such as ABCG2, which facilitate toxicant clearance (Merzendorfer 2014). The alterations in abundance of proteins involved in immune function, neurotoxicity, and oxidative stress—observed across 3- and 7-day exposure durations—suggest that CPF toxicity extends far beyond detoxification, leading to systemic cellular damage and potentially long-term physiological consequences for bat populations.

Overall, based on the function of the detected proteins and on those pathways that were enriched, we found that CPF exposure could affect metabolism, DNA integrity, cellular stress responses, and immune function in bats even after a short exposure time. Even though DAPs after 3 days of exposure did not retain statistical significance after applying the B–H correction, they remain valuable for generating hypotheses and providing insights into the potential physiological pathways affected by short exposure to CPF. For example, after 3 days of exposure bats showed downregulation in proteins associated with mitochondrial function, suggesting early impairment in energy metabolism, with a compensatory increase of metabolic regulators that could aid energy demands and detoxification at later exposure times. CPF may also be inducing genomic instability, evidenced by enriched pathways related to telomere maintenance (CPF-3d) and the downregulation of DNA repair proteins (CPF-7d) such as LIG1, which may increase mutation rates and contribute to cellular dysfunction. This mechanism could underlie the genotoxic stress previously reported in big brown bats exposed to CPF (Sandoval-Herrera et al. 2023), as disruptions in DNA replication and repair are known to lead to micronuclei formation (Krupina et al. 2021). Additionally, we found evidence of neurotoxicity in both treatment groups, with a progressive dysregulation of proteins associated with synaptic stability and neurodevelopment, as well as an enrichment of proteins involved in processes such as “behavior” and “locomotion.” These findings align with the well-documented neurotoxic effects of CPF and underscore the sensitivity of peripheral blood markers in detecting neurotoxicity. These findings suggest that CPF can disrupt multiple physiological processes in bats, with early metabolic impairment and DNA damage potentially leading to cascading effects in other systems and cellular homeostasis.

We observed significant changes in several proteins that participate directly or indirectly in processes related to immune function. Some of the most relevant pathways include interferon signaling, pathogen detection, cytokine regulation, and white blood cell function, as well as scaffold proteins that modulate immune responses indirectly. We detected signs of early immunosuppression at 3 days of exposure, marked by reduced levels of proteins mediating cell apoptosis (GZMA) and inflammation (TNIP3). By 7 days, dysregulation intensified, with impairment of the complement system (decreased MASP1), a crucial defense mechanism against pathogens (Dobó et al. 2014), previously reported to be important for bats' immunity (Becker et al. 2022). In addition to these proteins that are directly involved in immune processes, CPF-induced changes in proteins like ZC3H6, DUSP15, and XDH could indirectly impair immune response by increasing oxidative stress, specifically accelerating the production of reactive oxygen species (ROS)—a commonly reported CPF toxic effect (Dominah et al. 2017; Ubaid Ur Rahman et al. 2021). Other indirect effects included disruptions in DNA maintenance (e.g., downregulated POT1, TERF2, which protect telomeres) and endosomal trafficking (e.g., upregulated SNX33), potentially impairing cellular apoptosis and inflammation. Compromised genome integrity could undermine the exceptional longevity and healthy aging observed in bats, which has been linked to their unique telomere maintenance and DNA repair mechanisms (Foley et al. 2018; Huang et al. 2019; Cooper et al. 2024).

Pesticide-induced immunosuppression can disrupt disease dynamics in bats by increasing their susceptibility to infections and potentially altering their ability to transmit pathogens. While several studies have linked environmental toxicants to changes in bat-associated microbial diversity (Korine et al. 2017; Mehl et al. 2021; Lobato-Bailón et al. 2023), the direct relationship between these stressors, immunotoxicity, and their impact on parasite diversity or pathogen transmission remains poorly understood. Lilley et al. (2013) provided the only direct evidence of pesticide-induced immunotoxicity, showing that organochlorine pesticide bioaccumulation reduced complement system activity in Daubenton's bats (*Myotis daubentonii*). Similarly, Korine et al. (2017) observed increased ectoparasite loads in bats foraging over polluted waters, suggesting that contaminants may weaken immune defenses, increasing susceptibility to infections. Immunotoxicity is particularly concerning for bat conservation in light of current bat diseases, particularly white-nose syndrome (WNS), a fungal disease caused by *Pseudogymnoascus destructans* that has decimated bat populations in North America (Frick et al. 2010). The fungus disrupts hibernation, causing excessive energy depletion and often death (Davy et al. 2017). Since hibernation already suppresses immune function, pesticide-induced immunosuppression could further exacerbate WNS severity, accelerating its spread and increasing mortality rates (Warnecke et al. 2012; Eskew and Todd 2013). Despite these findings, the direct causal mechanisms linking toxicant exposure to altered pathogen dynamics remain a critical gap in our understanding of bat health and disease ecology. Recent efforts to tackle this complex question have incorporated mechanistic models and conceptual frameworks to propose the underlying mechanisms (Sánchez et al. 2020; Becker et al. 2024).

Bats have evolved unique adaptations that enhance their innate antiviral responses relative to most other mammals. For instance, there are well-documented differences in key components of the interferon pathway, the first line of defense against viral infections (Ahn et al. 2016; Ahn et al. 2019; Clayton and Munir 2020; Zhang and Irving 2023). This pathway drives the expression of interferon-stimulated genes (ISGs), which influence how effectively an individual controls a virus, ultimately shaping the duration and intensity of viral shedding. We found that CPF exposure induced changes in the abundance of several interferon-related proteins, including GIMAP8, GZMB, TLR7, and ZNFX1. These changes could weaken viral detection (e.g., reduced ZNFX1) or lead to excessive immune activation (e.g., increased GZMB and TLR7), potentially resulting in chronic inflammation and reduced viral tolerance. Most notably, CPF significantly reduced BST2 (tetherin), a protein that has been shown in bats to effectively restrict the release of viruses such as Ebola, HIV, and Nipah (Neil et al. 2007; Hoffmann et al. 2019). BST2 tethers viral particles to infected cells, inhibiting their release and further spread (Zhang and Irving 2023), and its downregulation may compromise bats’ exceptional viral resistance. Another key adaptation in bats' immunity is the precise regulation of inflammatory cytokines, preventing excessive immune responses and consequent pathogenesis (Banerjee et al. 2017, 2020). This balance is largely maintained by negative regulators of innate immunity, such as MyD88. We found that CPF exposure reduced the abundance of SF3A, an mRNA splicing complex essential for producing MyD88, a key negative regulator of TLR signaling (De Arras and Alper 2013). Therefore, this pesticide-induced decrease in SF3A could impair immune regulation, leading to heightened inflammation and potentially increased risk of disease in bats.

## Conclusions

Our study provides compelling evidence that CPF exposure disrupts critical physiological processes in bats, affecting immune function, DNA maintenance, and cellular metabolism. These alterations may weaken immune resilience, increase susceptibility to infections, and reduce overall fitness, potentially compromising bats' unique pathogen resistance. The cumulative effects of pesticide exposure, especially after prolonged exposure, combined with other environmental stressors, may further exacerbate immunosuppression and increase disease risk. Given bats' role as potential reservoirs for zoonotic pathogens, CPF exposure may have broader implications for disease transmission dynamics, and human health.

Pesticide use associated with agriculture expansion is a threat to bat populations that has been overlooked and that can represent a major conservation problem in agriculture-dominant regions. Bats are keystone species in ecosystems worldwide, contributing to pest control, seed dispersal, and pollination. Compromises to their ecological function and survival could therefore have far-reaching implications for ecosystem health. By integrating proteomics into ecoimmunology, we provide a powerful framework for understanding how environmental contaminants impact wildlife at a molecular level. Advances in -omics technologies have opened new avenues for research, allowing for a deeper exploration of these effects, especially when combined with *in vitro* immune response assays (e.g., microbial killing assays and antigen challenges) and traditional immune biomarkers. Future studies should bridge the gap between molecular disruptions and functional outcomes by linking proteomic alterations to phenotypic changes, epidemiological consequences, and long-term population health. Understanding these impacts is essential for guiding conservation strategies and mitigating the growing anthropogenic threats that wildlife face in an increasingly human-altered world.

## Author contributions

N.S.H.: conceptualization, formal analysis, investigation, visualization, writing original draft, and reviews. L.L.J.; conceptualization, data curation, methodology, investigation, review, and editing; P.A.F.; funding acquisition, supervision, review, and editing; D.B.D.S.; conceptualization, funding acquisition, supervision, review, and editing, resources, and methodology; K.C.W.; conceptualization, funding acquisition, supervision, review, and editing.

## Supplementary Material

icaf121_Supplemental_File

## Data Availability

Data are available via ProteomeXchange with identifier PXD064919 (https://panoramaweb.org/TV3y3R.url).

## Notes

From the symposium “Applied ecoimmunology in wildlife health and conservation" presented at the annual meeting of the Society for Integrative and Comparative Biology, January 3-7th , 2025.
