# Supplementary material for: Systemic Effects of Pesticides on Insectivorous Bats: A Proteomics Approach
Source: Integr Comp Biol. 2025 Jul 1;65(6):1810–25. doi: 10.1093/icb/icaf121 (PMC12690467; doi:10.1093/icb/icaf121)
Supplement: icaf121_Supplemental_File [file icaf121_supplemental_file.pdf]

Acquisition Method Report

Acquisition Method Info

**Method Name**	Agilent_training_peptides_slope4.m
**Method Path**	D:\MassHunter\Methods\peptides\Agilent_training_peptides_slope4.m
**Method Description**	

Device List

- Multisampler
- Binary Pump
- Column Comp.
- Q-TOF

TOF/Q-TOF Mass Spectrometer

**Component Name**	MS Q-TOF	**Component Model**	G6545A
**Ion Source**	Dual AJS ESI	**Stop Time (min)**	No Limit/As Pump
**Can wait for temp.**	Enable	**Fast Polarity**	False
**MS Abs. threshold**	500	**MS Rel. threshold(%)**	0.010
**MS/MS Abs. threshold**	5	**MS/MS Rel. threshold(%)**	0.010

Time Segments

Time Segment #	Start Time (min)	Diverter Valve State	Storage Mode	Ion Mode
1	0	MS	Both	Dual AJS ESI

Time Segment 1

Acquisition Mode AutoMS2

**MS Min Range (m/z)**	200
**MS Max Range (m/z)**	3000
**MS Scan Rate (spectra/sec)**	3.00
**MS/MS Min Range (m/z)**	50
**MS/MS Max Range (m/z)**	3000
**MS/MS Scan Rate (spectra/sec)**	2.00
**Isolation Width MS/MS**	Medium (~4 amu)
**Decision Engine**	Native

Ramped Collision Energy

Charge	Slope	Offset
All	4	2

Auto MS/MS Preferred/Exclude Table

Mass	Delta Mass (ppm)	Charge	Type	Retention Time (min)	Delta Ret. Time (min)	Isolation Width	Collision Energy
921.9686	100	1	Exclude	0		Narrow (~1.3 amu)	

Precursor Selection

**Max Precursors Per Cycle**	10
**Threshold (Abs)**	500
**Threshold (Rel)(%)**	0.010
**Precursor abundance based scan speed**	Yes
**Target (counts/spectrum)**	25000.000
**Use MS/MS accumulation time limit**	Yes
**Use dynamic precursor rejection**	No
**Purity Stringency (%)**	100.000
**Purity Cutoff (%)**	30.000
**Isotope Model**	Peptides
**Active exclusion enabled**	Yes
**Active exclusion excluded after (spectra)**	2
**Active exclusion released after (min)**	0.20
**Sort precursors**	By abundance only

Static Exclusion Ranges

StartMZ	EndMZ
25	300

Charge State Preference

Selected Charges
2
3
>3

Instrument Parameters

Parameter	Value
Gas Temp (°C)	325
Gas Flow (l/min)	8
Nebulizer (psig)	35
SheathGasTemp	350
SheathGasFlow	11

Scan Segments

Scan Seg #	Ion Polarity
1	Positive

Scan Segment 1

Scan Source Parameters

Parameter	Value
VCap	4500
Nozzle Voltage (V)	1000
Fragmentor	180
Skimmer1	65
OctopoleRFPeak	750

ReferenceMasses

**Ref Mass Enabled**	Disabled
**Ref Nebulizer (psig)**	

Chromatograms

Chrom Type	Label	Offset	Y-Range
TIC	TIC	15	10000000
TIC	TIC	15	10000000

Name: Multisampler — Module: G7167A

Sampling Speed

**Draw Speed**	100.0 µL/min
**Eject Speed**	400.0 µL/min
**Wait Time After Drawing**	1.2 s

Injection

**Needle Wash Mode**	Standard Wash
**Injection Volume**	2.00 µL

Standard Needle Wash

**Needle Wash Mode**	Flush Port
**Duration**	10 s

High Throughput

**Injection Valve to Bypass for Delay Volume Reduction**	No
**Sample Flush-Out Factor**	5.0

Overlapped Injection

**Overlap Injection Enabled**	No

Needle Height Position

**Draw Position Offset**	-1.0 mm
**Use Vial/Well Bottom Sensing**	No

Stop Time

**Stoptime Mode**	No Limit

Post Time

**Posttime Mode**	Off

Name: Binary Pump — Module: G7112B

**Flow**	0.000 mL/min
**Use Solvent Types**	Yes
**Low Pressure Limit**	0.00 bar
**High Pressure Limit**	400.00 bar
**Maximum Flow Gradient**	100.000 mL/min²

Stroke A

**Automatic Stroke Calculation A**	Yes

Stroke B

**Automatic Stroke Calculation B**	Yes

Stop Time

**Stoptime Mode**	Time set
**Stoptime**	50.00 min

Post Time

**Posttime Mode**	Off

Solvent Composition

	Channel	Solvent 1	Name 1	Solvent 2	Name 2	Selected	Used	Percent (%)
**1**	A	H20		H20		Ch. 1	Yes	98.0 %
**2**	B	premixed ACN(95%) - H2O(5%)		ACN		Ch. 1	Yes	2.0 %

Timetable

	Time (min)	A (%)	B (%)	Flow (mL/min)
**1**	0.00 min	98.0 %	2.0 %	0.100 mL/min
**2**	2.00 min	98.0 %	2.0 %	0.100 mL/min

	Pressure (bar)
1	400.00 bar
2	400.00 bar

	Time (min)	A (%)	B (%)	Flow (mL/min)
3	27.00 min	60.0 %	40.0 %	0.100 mL/min
4	32.00 min	40.0 %	60.0 %	0.100 mL/min
5	32.01 min	15.0 %	85.0 %	0.100 mL/min
6	37.00 min	15.0 %	85.0 %	0.100 mL/min
7	37.01 min	98.0 %	2.0 %	0.100 mL/min

Name: Column Comp. **Module:** G7116A

Left Temperature Control

Temperature Control Mode Temperature Set

Temperature 40.0 °C

Enable Analysis Left Temperature

Enable Analysis Left Temperature On Yes

Enable Analysis Left Temperature Value 1.0 °C

Left Temp. Equilibration Time 0.0 min

Right Temperature Control

Right temperature Control Mode Temperature Set

Right temperature 40.0 °C

Enable Analysis Right Temperature

Enable Analysis Right Temperature On Yes

Enable Analysis Right Temperature Value 0.8 °C

Right Temp. Equilibration Time 0.0 min

Enforce column for run

Enforce column for run enabled No

Stop Time

Stoptime Mode As pump/injector

Post Time

Posttime Mode Off

Timetable

Valve Position Position 2 (Port 1 -> 2)

Position Switch After Run Do not switch

	Pressure (bar)
**3**	400.00 bar
**4**	400.00 bar
**5**	400.00 bar
**6**	400.00 bar
**7**	400.00 bar
